# Association between dietary inflammatory index and metabolic syndrome: Analysis of the NHANES 2005–2016

**DOI:** 10.3389/fnut.2022.991907

**Published:** 2022-10-06

**Authors:** Xiaochen Zhang, Yinpei Guo, Nan Yao, Ling Wang, Mengzi Sun, Xiaomeng Xu, Huanshuai Yang, Yang Sun, Bo Li

**Affiliations:** ^1^School of Public Health, Jilin University, Changchun, China; ^2^Department of Epidemiology and Biostatistics, School of Public Health, Jilin University, Changchun, China

**Keywords:** dietary inflammatory index, metabolic syndrome, association analysis, hierarchical analysis, NHANES

## Abstract

**Objective:**

Metabolic syndrome (MetS) is a global problem that increasingly violates human health and quality of life. We explored the relationship between dietary inflammatory potential represented by dietary inflammatory index (DII) and the occurrence of MetS to provid data support for the prevention of it through dietary structure intervention.

**Methods:**

The data was come from National Health and Nutrition Examination Survey 2007–2018, including demographic, dietary, questionnaire variables and laboratory indicators. MetS was defined according to the criteria proposed by the American Endocrine Association (ACE) and the American Society of Clinical Endocrinology (ACCE). DII was calculated using the scoring method established by Shivappa. We divided DII scores into 4 quartiles, the chi-square test was used to compare the variable difference between DII quartiles groups. A logistic regression model was used to analyze the association between DII and MetS. We also performed subgroup analysis. A generalized linear regression model was used to explore the association of DII level and the levels of seven MetS related biochemical indicators.

**Results:**

The final sample size was 8,180, and the DII scores of the subjects were −5.50 to 5.22. The proportions of men, young people, non-Hispanic blacks, poor people, smokers, and MetS patients in the Q1–Q4 DII quantiles groups were gradually increased. The risk of MetS in the Q4 group which had highest dietary inflammatory degree was 1.592 (1.248, 2.030) times higher than that in the Q1 group, respectively (*P* < 0.001). After subgroup analysis, women, youth, non-smokers and alcohol drinkers were found to be more sensitive to the dietary inflammation. Then we found that the level of DII was significantly positively correlated with waist circumference (WC), body mass index (BMI), triglyceride (TG), systolic blood pressure (SBP) and diastolic blood pressure (DBP), but negatively correlated with high density lipoprotein cholesterol (HDL-C).

**Conclusions:**

In the research subjects, the degree of dietary inflammation was associated with the occurrence of MetS and significantly affected WC, BMI, blood pressure, and blood lipid levels. It is necessary to conduct investigations and early dietary interventions for women and young people to prevent the occurrence of chronic metabolic diseases.

## Introduction

Metabolic syndrome (MetS) is a complex of cardiovascular disease and diabetes related risk factors, including hypertension, impaired fasting glucose, abdominal obesity and dyslipidemia (1–3). MetS is becoming a global problem that increasingly violates human health and quality of life, and the risk of its occurrence increases with age (4, 5). In most developed countries, the prevalence of MetS in adults is about 20–30%, however in developing countries and regions, the prevalence of it is lower but nowadays is approaching or even surpassing that in developed countries (6). In addition, studies have found that these risk factors increasingly begin in childhood and adolescence and are closely associated with the diagnosis of various chronic diseases in adulthood (7). Therefore, the gradual rejuvenation, high prevalence of MetS and its association with the diagnosis of other chronic diseases all suggest its clinical severity. How to detect, diagnose and intervene early MetS is a topic that people are gradually paying attention to.

The production of MetS seems to originate from excessive central obesity, leading to dysfunctional release of adipocytes in the body, resulting in changes in the levels of inflammatory factors and triggering a systemic anti-inflammatory state (8). Some studies have suggested that the early manifestations of MetS are mainly insulin resistance. In addition to leading to the occurrence of type 2 diabetes, it will further affect blood pressure and high-density lipoprotein cholesterol levels, the latter as a trigger for cardiovascular disease. It is an important risk factor, and its pathological changes will lead to the final diagnosis of a series of chronic metabolic diseases (9–11). Therefore, obesity may be the initial cause of MetS, thus weight loss, central obesity reduction, sedentary reduction and the formation of a healthy diet seem to be important interventions to effectively reduce the occurrence of metabolic abnormalities (12).

A systemic inflammatory state is an important pathological process prior to the development of various metabolic diseases, and studies have shown that the infiltration of macrophages and other immune cells observed in adipose tissue, liver, muscle and pancreas is associated with a shift in cell populations from anti-inflammatory to pro-inflammatory state. These tissues are important sites of inflammation in obese people. Therefore, activation of the immune system is closely related to obesity-related insulin resistance, type 2 diabetes and even the pathogenesis of other metabolic diseases (13).

The anti-inflammatory diet can reverse the systemic inflammatory state to a certain extent and slow down the development of various metabolic diseases. These changes can be explored by evaluating the levels of inflammatory factors or by long-term cohort trials. Previous studies have suggested that Western dietary patterns are associated with increased levels of high-sensitivity CRP, especially in adolescents; while reducing the intake of high-inflammatory foods such as red meat and refined grains significantly reduces the levels of inflammatory factors (14, 15). In contrast, a Mediterranean diet reduces systemic inflammation and protects the body from MetS (16).

The dietary inflammation index (DII^®^) is a scientific evaluation method that uses the intake values of various food parameters of the research subjects to calculate the degree of individual dietary inflammation level used to explore the role of high inflammatory diet in the occurrence and development of metabolic diseases. The score was established by Nitin Shivappa and group in 2009 and refined in 2014, eventually incorporating 45 food parameters that include nutrients and bio-active compounds to assess the inflammatory potential of an individual's diet and linking to a global reference value for 11 populations around the world for featured use across regions and populations (17).

At present, people are increasingly interested in dietary patterns for the prevention of chronic diseases (18). A number of studies on dietary structure and metabolic diseases are gradually being carried out. Many scholars have concluded that certain positive role of nutrients in improving insulin sensitivity and reduce blood sugar levels (19, 20). However, the relationship between the degree of dietary inflammation and MetS is still controversial, some studies have not found a clear relationship between this two (21, 22). Therefore, in order to further explore whether the occurrence of MetS is related to the degree of dietary inflammation in individuals, this study completed the association analysis between DII and MetS occurrence using national representative data from 2007 to 2018 in the United States, aimed to provide data support for the reduce chronic MetS through dietary structure adjustment.

## Materials and methods

### Data sources

This study was based on demographic, dietary, laboratory, and questionnaire databases from the 2007–2018 National Health and Nutrition Examination Survey (NHANES).

### The establishment of the database

#### Data collection and preprocessing

(1) Demographic data: gender, age, race, marital status, education level, pregnancy status and household income-to-poverty ratio (PIR), taking 1.3 as the limit, subjects with PIR <1.3 were defined as poor people (23).
(2) Dietary data: The average value of the nutrient intake in the dietary database on the first day and the dietary database on the second day was used as the final individual nutrient intake level. When the dietary data of 1 day was missing, the average value was replaced by another day value. Total energy and 27 food parameters available in NHANES were included, including 6 pro-inflammatory food parameters: total fat, saturated fatty acids, protein, carbohydrates, cholesterol, and vitamin B12, and 21 anti-inflammatory parameters: monounsaturated fatty acids, polyunsaturated fatty acids, dietary fiber, omega-3 fatty acids, omega-6 fatty acids, beta-carotene, alcohol, caffeine, niacin, vitamin A, thiamine (Vitamin B1), riboflavin (Vitamin B2), Vitamin B6, vitamin C, vitamin D, vitamin E, iron, magnesium, zinc, selenium and folic acid.
(3) Measurement and laboratory data: waist circumference (WC), body mass index (BMI), systolic blood pressure (SBP), diastolic blood pressure (DBP), triglyceride (TG), high density lipoprotein cholesterol (HDL-C) and fasting blood glucose (FPG).
(4) Questionnaire data: family history of diabetes, history of taking insulin, history of taking antihypertensive drugs, history of taking lipid-lowering drugs; coronary heart disease history, tumor history, heart attack history and other major diseases as well as physical activity related indicators. According to World Health Organization guidelines (24), this study defined samples with more than 149 min of moderate physical activity or more than 74 min of heavy physical activity or more than 599 min of metabolic equivalents (MET) per week as active, and others as inactive.

#### Inclusion and exclusion criteria

The initial total sample size was 59,842. Samples aged 20 to 64 were included; (1) pregnant women; (2) samples with severe diseases such as malignant tumors and coronary heart disease; (3) the average total dietary energy intake for 2 days was too high or too high. Samples with extreme energy intakes (females <500 kcal/d or >5,000 kcal/d; males <500 kcal/d or >8,000 kcal/d) (25); (4) samples with missing main indicators and dietary data were excluded. The final sample size was 8,180.

#### Definition of MetS

According to the criteria proposed by the American Endocrine Association (ACE) and the American Society of Clinical Endocrinology (ACCE) (1), MetS was considered to occur when 3 of the following 5 conditions are present: (1) Elevated WC (≥88 cm for females, ≥102 cm for males), (2) elevated TG (≥150 mg/dL) or drug-treated TG, (3) low HDL-C (<40 mg/dL for males and <50 mg/dL for females) or medication for low HDL-C, (4) elevated blood pressure (SBP ≥130 mm Hg or DBP ≥85 mm Hg or both) or antihypertensive medication, (5) elevated FPG (≥100 mg/dL) or drug-treated blood glucose.

### The calculation of DII

DII was calculated according to the scoring method established and improved by Nitin Shivappa et al. (17). Based on the established algorithm, we used total energy to adjust the intake level of 27 food parameters in order to obtain the corresponding food parameter level per 1,000 kcal (26–28). Total energy was used to adjust the intake level of 27 food parameters in order to obtain the corresponding food parameter level per 1,000 kcal. Then used the mean value and standard deviation of each parameter from NHANES 2007–2008 to perform z-transformation on the corresponding nutrients, then ranked the z-transformation scores of each parameter and performed percentile transformation to obtain the percentile of each sample, and finally doubled and then subtracted by 1 to obtain the adjusted parameter levels. Finally, individual DII score was obtained by multiplying the final adjusted levels of parameters by their respective inflammatory effect scores and adding them together (29, 30). A higher DII score represented a higher degree of dietary inflammation, and a lower DII score represented a lower degree of dietary inflammation (31).

### Statistical analysis

Qualitative data were expressed by the number of cases and the composition ratio, and the DII scores of the subjects were divided into 4 quartiles. The chi-square test was used for the comparison between groups of different DII quartiles, and the linear trend test was performed using the median of each quantile array as the independent variable. A logistic regression model was used to analyze the association between DII and MetS. The subjects were grouped according to gender, age, smoking status, and alcohol consumption status, and the DII dichotomy was used as the independent variable x for subgroup analysis to identify sensitive groups. A generalized linear regression model was used to explore the association of DII levels with the levels of seven MetS related biochemical indicators. Model 1 was a rough model, only controlling for age, gender, and race. Model 2 added controls for education, marital status, and PIR. In the final model, model 3 further controlled for smoking, alcohol consumption, family history of diabetes, and physical activity. IBM SPSS 24.0 Statistical Software was used for analysis, and *P* < 0.05 was considered statistically significant.

## Results

The sample size of this study was 8,180, and the DII scores of all subjects were between −5.50 and 5.22. After the DII quartiles were grouped, the Q1 group represented the lowest dietary inflammatory group, −5.50≤DII <-1.39; the Q2 group represented the lower dietary inflammation group, −1.39≤DII <-0.01; the Q3 group represents the higher dietary inflammation group, −0.01≤DII <1.25; the Q4 represents the highest dietary inflammation group, 1.25≤DII <5.22.

Table 1 showed the distribution of all variables in 4 DII quartile groups. Except for family history, alcohol consumption and physical activity, the distribution of gender, age, race, marriage, education, poverty, smoking status was statistically different in 4 groups. Among them, the proportion of males in the Q1–Q4 groups gradually increased (P-trend < 0.05), and the proportion of females gradually decreased. The proportion of youth in the four quantile groups gradually increased, and the opposite was true for middle-aged people. The proportion of non-Hispanic blacks increased most significantly in the Q1–Q4 groups. The proportion of married people, people with college education and above in the quartile group gradually decreased. The proportion of poor people and smokers, as well as MetS patients in the Q1–Q4 groups gradually increased.

**Table 1:** The distribution of the basic situation of the subjects in 4 DII quartile groups [*n* (%)].

**Variable**		**Q1^a^** **(*n* = 1,955)**	**Q2^a^** **(*n* = 2,069)**	**Q3^a^** **(*n* = 2,016)**	**Q4^a^** **(*n* = 2,140)**	***χ^2^***	***P***	***P-trend***
Gender						94.1	**<0.001**	**<0.001**
	Male	775 (42.4%)	952 (46.1%)	1,042 (54.2%)	1,206 (55.8%)			
	Female	1,180 (57.6%)	1,117 (53.9%)	974 (45/8%)	934 (44.2%)			
Age						127.7	**<0.001**	**<0.001**
	Middle-aged (≥45)	1,032 (50.7%)	995 (48.0%)	858 (41.6%)	759 (34.1%)			
	Youth (<45)	923 (49.3%)	1,074 (52.0%)	1,158 (58.4%)	1,381 (65.9%)			
Race						195.0	**<0.001**	**<0.001**
	Non-hispanic white	707 (64.8%)	770 (62.9%)	779 (64.7%)	938 (65.0%)			
	Non-hispanic black	281 (6.7%)	332 (8.2%)	450 (12.7%)	613 (16.3%)			
	Other	407 (13.0%)	309 (10.2%)	212 (6.7%)	144 (5.4%)			
	Mexican and hispanic	560 (15.5%)	658 (18.7%)	575 (15.9%)	445 (13.4%)			
Marital status						143.9	**<0.001**	**<0.001**
	Other including divorced	441 (20.5%)	530 (24.7%)	538 (24.4%)	656 (28.3%)			
	Married	1,132 (59.0%)	1,134 (57.4%)	993 (50.8%)	877 (42.6%)			
	Never married	382 (20.5%)	405 (17.9%)	485 (24.8%)	607 (29.1%)			
Education level						385.9	**<0.001**	**<0.001**
	Junior high school or below	291 (8.7%)	435 (13.1%)	435 (13.7%)	535 (19.7%)			
	High school	293 (12.9%)	403 (20.2%)	403 (26.1%)	631 (31.6%)			
	College or above	1,371 (78.4%)	1,231 (66.7%)	1,117 (60.2%)	974 (48.7%)			
PIR level						145.8	**<0.001**	**<0.001**
	Poverty (<1.3)	484 (16.2%)	628 (22.3%)	668 (24.8%)	908 (32.7%)			
	Non-poverty (≥1.3)	1,471 (83.8%)	1,441 (77.7%)	1,348 (75.2%)	1,232 (67.3%)			
Family history of diabetes						16.4	0.097	0.130
	Yes	792 (36.6%)	877 (40.1%)	840 (39.1%)	925 (43.0%)			
	No	1,163 (63.4%)	1,192 (59.9%)	1,176 (60.9%)	1,215 (57.0%)			
Smoke						167.0	**<0.001**	**<0.001**
	Yes	645 (35.3%)	770 (38.8%)	867 (42.8%)	1,142 (54.7%)			
	No	1,310 (64.7%)	1,299 (61.2%)	1,149 (57.2%)	998 (45.3%)			
Alcohol consumption						18.9	0.072	0.375
	Yes	1,246 (72.4%)	1,331 (68.2%)	1,403 (74.1%)	1,522 (72.9%)			
	No	709 (27.6%)	738 (31.8%)	613 (25.9%)	618 (27.1%)			
Physical activity						18.7	0.114	0.154
	Yes	437 (27.6%)	407 (23.9%)	412 (24.7%)	431 (21.6%)			
	No	1,518 (72.4%)	1,662 (76.1%)	1,604 (75.3%)	1,709 (78.4%)			
Metabolic syndrome						20.7	**0.049**	**0.029**
	Yes	690 (32.5%)	766 (36.4%)	839 (39.5%)	830 (36.9%)			
	No	1,265 (67.5%)	1,303 (63.6%)	1,177 (60.5%)	1,310 (63.1%)			

^a^ Q1: −5.50 ≤ DII <-1.39, Q2:−1.39 ≤ DII <-0.01, Q3: −0.01 ≤ DII <1.25, Q4: 1.25 ≤ DII <5.22. Statistically significant *p*-values were shown in bold.

Table 2 showed the results of logistic regression analysis of the prevalence of DII and MetS. In the crude model 1, the risk of MetS in the Q4 group was 1.826 (1.433, 2.326) times that of the Q1 group. In models 2 and 3 with more variables controlled, the risk of MetS in the Q3 group was Q1 group was 1.646 (1.244, 2.177) times and 1.636 (1.220, 2.196) times, and the risk of MetS in Q4 group with the highest degree of dietary inflammation was 1.675 (1.316, 2.132) times and 1.592 (1.248, 2.030) times that of Q1 group, respectively.

**Table 2:** Logistic regression analysis of DII and metabolic syndrome.

**Model**	**Group**	***Waldχ2***	***P***	**OR (95% CI)**
Model 1^a^		27.804	**<0.001**	
	Q1			1
	Q2		**0.044**	1.298 (1.007, 1.673)
	Q3		**<0.001**	1.729 (1.314, 2.275)
	Q4		**<0.001**	1.826 (1.433, 2.326)
Model 2^b^		19.875	**<0.001**	
	Q1			1
	Q2		0.076	1.256 (0.976, 1.615)
	Q3		**0.001**	1.646 (1.244, 2.177)
	Q4		**<0.001**	1.675 (1.316, 2.132)
Model 3^c^		16.527	**0.001**	
	Q1			1
	Q2		0.128	1.221 (0.943, 1.579)
	Q3		**0.001**	1.636 (1.220, 2.196)
	Q4		**<0.001**	1.592 (1.248, 2.030)

^a^Model 1: Control variables: gender, age, race. ^b^ Model 2: Control variables: gender, age, race, education level, marital status, PIR level. ^c^ Model 3: Control variables: gender, age, race, education level, marital status, PIR level, family history of diabetes, smoking situation, alcohol situation, physical activity. Statistically significant *p*-values were shown in bold.

Table 3 showed the results of subgroup analysis of the association between DII and MetS in different gender, age, smoking, and alcohol consumption groups under Model 3. The results suggested that in females, the risk of developing MetS in the higher dietary inflammatory group was 1.478 (1.144, 1.910) times that of the lower dietary inflammatory group, while in males, the risk was 1.420 (1.112, 1.814) times, so it can be seen that females were more sensitive to dietary inflammation than males (1.478 > 1.420). Similarly, the young population was more sensitive than the middle-aged population. In the young population, the risk of MetS in the higher dietary inflammatory group was 1.506 (1.198, 1.892) times that of the lower dietary inflammatory group, while 1.405 (1.045, 1.888) times in middle-aged population. No significant association was found between dietary inflammation and MetS prevalence in smokers and non-drinkers. In non-smokers, the risk of MetS in the higher dietary inflammatory group was 1.656 (1.306, 2.101) times that of the lower dietary inflammatory group, and the risk in the alcohol consumption groups was 1.482 (1.183, 1.856) times. Therefore, non-smokers and drinkers were more sensitive to dietary inflammation.

**Table 3:** Subgroup analysis of the association of DII and MetS.

**Subgroup**		**Cases/subjects**	***Waldχ2***	***P***	**OR (95% CI)**
Gender	Male	(1,533/3,975)	8.132	**0.005**	1.420 (1.112, 1.814)
	Female	(1,592/4,205)	9.164	**0.003**	1.478 (1.144, 1.910)
Age	Youth (<45)	(1,178/4,536)	12.653	**0.001**	1.506 (1.198, 1.892)
	Middle-aged (≥45)	(1,947/3,644)	5.210	**0.025**	1.405 (1.045, 1.888)
Smoke	Yes	(1,464/3,424)	3.175	0.078	1.277 (0.972, 1.678)
	No	(1,661/4,756)	17.782	**<0.001**	1.656 (1.306, 2.101)
Alcohol consumption	Yes	(2,014/5,520)	12.061	**0.001**	1.482 (1.183, 1.856)
	No	(1,111/2,678)	3.187	0.077	1.388 (0.964, 1.997)

Statistically significant *p*-values were shown in bold.

Table 4 showed the relationship between DII and seven MetS related biochemical indicators under model 3. After generalized linear regression, it was found that the level of DII was significantly positively correlated with the levels of WC, BMI, TG, SBP and DBP, and was significantly correlated with the level of HDL-C. Among them, the association coefficient between TG and DII was the largest (β = 2.795), followed by the association between WC and DII (β = 0.881), the association coefficient between HDL-C and DII was −0.803, and the smallest association was between DBP and DII.

**Table 4:** Association between DII and various MetS related biochemical indicators.

**Dependent variable**	***S.E*.**	***Waldχ2***	***P***	**β (95% CI)**
WC	0.104	71.779	**<0.001**	0.881 (0.677, 1.084)
BMI	0.044	51.748	**<0.001**	0.318 (0.232, 0.405)
TG	0.915	9.338	**0.002**	2.795 (1.003, 4.588)
HDL-C	0.093	74.365	**<0.001**	−0.803 (−0.986, −0.621)
SBP	0.096	10.304	**0.001**	0.309 (0.120, 0.498)
DBP	0.073	7.588	**0.006**	0.202 (0.058, 0.346)
FPG	0.220	1.090	0.296	0.229 (−0.201, 0.660)

WC, waist circumference; BMI, body mass index; SBP, systolic blood pressure; DBP, diastolic blood pressure; TG, triglyceride; HDL-C, high density lipoprotein cholesterol; FPG, fasting blood glucose. Statistically significant *p*-values were shown in bold.

## Discussion

Based on the controversial results obtained by previous studies on the relationship between dietary inflammation and MetS (22, 32), we used data from NHANES to analyze the association between dietary inflammation and MetS and its related biochemical indicators and explored sensitive population.

The average age of subjects was 42.06 ± 12.90, the prevalence of MetS was 38.2%, and the DII scores of all subjects were between −5.50 and 5.22. In the study conducted also in the United States of the association between DII and brain MRI, the DII scores ranged from −4.47 to 4.06 (33), and the DII scores of another study of the association between DII and the occurrence of frail events were −5.65 to 3.70 (34), proving that the DII score distribution in this study is similar to other studies.

After dividing subjects into 4 DII quartiles groups, we compared the distributions of different variables. The results showed that the proportion of males, young people, non-Hispanic blacks, poor people, smokers, and MetS patients in the quartile group Q1–Q4 had a significant upward trend (P-trend < 0.05), indicating that the diets of these subjects were significantly more inflammatory. This may indeed be the case. Males, young people, and smokers tend to pay less attention to their own health cognition and good living habits than females, more mature middle-aged people and non-smokers who pursue a more refined quality of life. Therefore, males are more prone to various metabolic diseases than females (35, 36). At present, chronic diseases have also shown a clear trend of younger age, adolescents have gradually become an important group with a high incidence of chronic diseases (37). At the same time, adolescents are also an effective group for lifestyle intervention. The results of a calorie control intervention study showed that moderate calorie restriction for 2 years significantly reduced a variety of cardiometabolic risk factors in young people and non-obese adults (38). Black or poor people may have relatively no purpose in obtaining food and do not consider whether their diet is healthy or not. Research on these groups needs to be further carried out. The number of people who drink alcohol in this study was relatively large. Even though whether alcoholic beverages have a positive effect on the body is still controversial in different studies, a systematic review published in The Lancet clearly stated that the harm of alcohol to the human body is objective, and no change will occur even if low intake (39). Therefore, we controlled for alcohol use in our model and performed subgroup analysis in drinkers and non-drinkers, even though the distribution of alcohol consumption in the Table 1 did not differ significantly between the DII quartile groups.

The logistic regression results showed that after controlling for potentially influential variables, pro-inflammatory dietary patterns were still associated with the occurrence of MetS. The risk of Q4 group was 1.592 (1.248, 2.030) times that of Q1 group, which echoing many previous studies showing that the Western diet with high salt, high fat, refined carbohydrates and the pro-inflammatory diet were closely related to the occurrence and development of cardiovascular and cerebrovascular diseases, type 2 diabetes and MetS. This process is often accompanied by an increase in the level of inflammatory factors in the body and an increase in systemic chronic inflammation, because the latter is an important pathological basis for the occurrence of various metabolic diseases (18, 40). However, some studies had reached different conclusions, such as the national cross-sectional study in Luxembourg exploring the association of inflammatory diet and CRP level, the PONS (research on chronic non-communicable diseases in European high risk countries) study in Poland, and the association study between adult western dietary patterns and MetS in Lebanon did not reach significant conclusions (41–43). This may be due to some differences in terms of region, sample size, statistical period, lifestyle and ethnic characteristics of the study population, or because the study did not adopt uniform MetS diagnostic criteria. At present, there is no clear and authoritative explanation for the specific mechanism of inflammatory diet triggering MetS, but some studies have proposed that the dietary structure can lead to changes in the body's inflammatory level or oxidative stress pathway by regulating the intestinal flora, leading to the occurrence of obesity or insulin resistance (44, 45). In addition, some have also suggested that the influence of diet on inflammation may be related to heredity (46).

The results of subgroup analysis showed that the risk of MetS of females and young people were higher than that of males (1.478 > 1.420) or middle-aged people (1.506 > 1.405). Females were more sensitive to inflammatory diets, which may be associated with greater waist-to-hip ratio (WHR) values which is a measure of intra-abdominal obesity, strongly associated with inflammation, one study showed that greater WHR values were associated with higher DII scores (41). At the same time, this phenomenon may also be related to hormone levels. It has been reported that estrogen helps to change fat metabolism, thus promoting the occurrence of MetS (47). Therefore, it is extremely important to pay attention to chronic disease prevention in females. Consistent with previous findings, the young people population was more sensitive to an inflammatory diet, which may be associated with higher levels of TNF-α and IL-6 (48). In addition, many dietary and behavioral intervention trials have continuously confirmed that early intervention had a significant positive effect on reducing the diagnosis of chronic metabolic diseases in the middle-aged in the future (38, 49). Smoking as a well-known independent risk factor for chronic non-communicable diseases, and alcohol consumption as a bad lifestyle habit that endangers human life and health, both of them have been considered in this study. The results showed that DII was significantly associated with MetS in non-smokers and drinkers, but no significant association was found between DII and MetS in smokers, which was consistent with the results of related studies in Korea (31). Lifestyle especially dietary habits changes are the main therapeutic strategy for the treatment and management of MetS, and multiple investigations have shown that the Mediterranean diet significantly reduced BMI in obesity and child and adolescent suspected of being diagnosed with MetS (24, 50). Therefore, for the above-mentioned sensitive groups found in this study, we suggest early intervention with an anti-inflammatory diet.

We also explored the direct association between DII level and seven biochemical indicators used to judge the occurrence of MetS. The results showed that in addition to FPG, DII was significantly positively correlated with the levels of WC, BMI, TG, SBP, and DBP, while significantly negatively correlated with HDL-C level, which echoing several previous studies. A meta-analysis found that in the included observational study, higher DII scores were associated with a 1.81 cm increase in WC (51). Furthermore, higher DII scores have been reported to be prospectively associated with higher TG and lower HDL-C concentrations (32, 52, 53).

Even our positive results may provide important ideas for developing more effective dietary interventions and public health policies to reduce inflammation and promote human metabolic health, there are still some limitations. Our data came from a cross-sectional survey—NHANES, so there exists limitation for the prospective exploration of risk factors. In addition, we directly deleted the study subjects who did not measure biochemical indicators during exclusion, thus, there may be some influence on the generalization of the research results to the American population.

## Conclusion

In the research subjects, the pro-inflammatory diet was associated with the occurrence of MetS, and significantly affected WC, BMI, blood pressure, and blood lipid levels. It is necessary to conduct investigations and early dietary interventions for females and young people to prevent the occurrence of chronic metabolic diseases.

## Data availability statement

The raw data supporting the conclusions of this article will be made available by the authors, without undue reservation.

## Author contributions

BL, YG, and NY contributed to conception and design of the study. XZ, XX, HY, and YS organized the database. XZ and YG performed the statistical analysis. XZ wrote the first draft of the manuscript. YG, NY, LW, and MS wrote sections of the manuscript. All authors contributed to manuscript revision, read, and approved the submitted version.

## Conflict of interest

The authors declare that the research was conducted in the absence of any commercial or financial relationships that could be construed as a potential conflict of interest.

## Publisher's note

All claims expressed in this article are solely those of the authors and do not necessarily represent those of their affiliated organizations, or those of the publisher, the editors and the reviewers. Any product that may be evaluated in this article, or claim that may be made by its manufacturer, is not guaranteed or endorsed by the publisher.
